# Two New Secondary Metabolites from *Xylaria* sp. cfcc 87468

**DOI:** 10.3390/molecules19011250

**Published:** 2014-01-20

**Authors:** Fuqian Wang, Shishi Han, Song Hu, Yongbo Xue, Jianping Wang, Hongfeng Xu, Lu Chen, Geng Zhang, Yonghui Zhang

**Affiliations:** 1 Department of Pharmacy, Wuhan First Hospital, Wuhan 430022, Hubei, China; E-Mails: wangfuqian.c@163.com (F.W.); huyaoshi@sina.com.cn (S.H.); xhf991030@tom.com (H.X.); chenglu19810620@163.com (L.C.); 2 Hubei Key Laboratory of Natural Medicinal Chemistry and Resource Evaluation, School of Pharmacy, Tongji Medical College, Huazhong University of Science and Technology, Wuhan 430030, Hubei, China; E-Mails: mshss880910@163.com (S.H.); yongboxue@mail.hust.edu.cn (Y.X.); jpwang1001@163.com (J.W.)

**Keywords:** *Xylaria*, isocoumarin glycoside, phenylethanol glycoside, steroids

## Abstract

A new isocoumarin glycoside, 3*R*-(+)-5-*O*-[6'-*O*-acetyl]-*α*-d-glucopyranosyl-5-hydroxymellein (**1**), and a new phenylethanol glycoside, (−)-phenylethyl-8-*O*-*α*-l-rhamno-pyranoside (**2**), were isolated from the ethyl acetate extract of the fungus *Xylaria* sp. cfcc 87468, together with five known steroids, *β*-sitosterol (**3**), stigmast-4-en-3-one (**4**), ergosterol (**5**), (22*E*)-cholesta-4,6,8(14),22-tetraen-3-one (**6**), and 4*α*-methyl- ergosta-8(14),24(28)-dien-3*β*-ol (**7**). The structures of compounds **1** and **2** were elucidated by MS, extensive 1D and 2D NMR spectroscopy, and the circular dichroism (CD) spectroscopy.

## 1. Introduction

**Figure 1:**
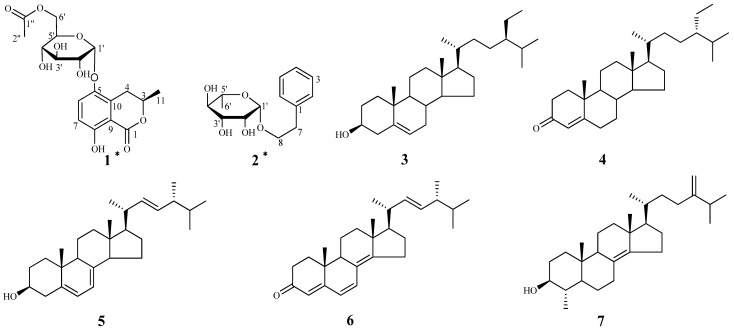
Chemical structures of compounds **1**–**7** from *Xylaria* sp. cfcc 87468.

Endophytes have proved to be an excellent source of new bioactive molecules [1,2]. The endophytic fungi of the genus *Xylaria* produce many types of secondary metabolites [3,4]. Isocoumarins are metabolites of limited distribution, which occur in bacteria, fungi and lichen [5]. The most recent article on the genus *Xylaria* described the isolation of *cis*-(3*R*,4*R*)-5-carbomethoxy-4-hydroxymellein from the fungus *Xylaria* sp. PSU-G12 [6], however, the secondary metabolites of the *Xylaria* sp. cfcc 87468 have not been investigated to date. As part of our ongoing efforts to find new bioactive natural products from the genus *Xylaria* sp. cfcc 87468, the chemical constituents of the EtOAc extract of *Xylaria* sp. cfcc 87468 cultures were investigated. This work resulted in the isolation of a new isocoumarin glycoside, a new phenylethanol glycoside, and five known steroids. In this paper, we describe the isolation and structure elucidation of these two new compounds, 3*R*-(+)-5-*O*-[6'-*O*-acetyl]-*α*-d-glucopyranosyl-5-hydroxymellein (**1**) and (−)-phenylethyl-8-*O*-*α*-l-rhamnopyranoside (**2**), and the five known compounds **3**–**7** (Figure 1).

## 2. Results and Discussion

Compound **1** was obtained as a colorless amorphous gum. 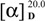 +60 (*c* = 1.33, CH_3_OH). A molecular formula of C_18_H_22_O_10_ was assigned based on the interpretation of HRESIMS peak at *m/z* 421.1097 [M+Na]^+^ (calcd. 421.1105). Its IR spectrum showed characteristic hydroxyl group (3397 cm^–1^), and two ester carbonyl group (1676 and 1737 cm^–1^) absorptions. The ^1^H-NMR data of **1** (Table 1) showed two aromatic proton signals at *δ*_H_ 6.83 (d, *J* = 9.2 Hz, 1H) and 7.45 (d, *J* = 9.2 Hz, 1H), two methyls at *δ*_H_ 1.98 (s, 3H) and 1.52 (d, *J* = 6.3 Hz, 3H), and an oxygenated proton at *δ*_H_ 5.35 (d, *J* = 3.7 Hz, 1H). The ^13^C-NMR and DEPT spectra of compound **1** displayed 18 carbon signals, including two methyls, two methylenes (one oxygenated methylene), six methines, six aromatic carbons (four quaternary carbons), and two ester carbonyl groups. One set of proton signals at *δ*_H_ 3.3–4.3, 5.35, and their corresponding carbons resonating at *δ*_C_ 64.9, 71.9, 72.1, 73.2, 74.8, and 100.5, suggested the presence of a hexosyl sugar moiety in the molecule. The ^1^H-NMR spectrum exhibited protons signals at *δ*_H_ 2.69 (dd, *J* = 17.0, 11.7 Hz, H-4a), 3.46 (dd, *J* = 17.0, 3.3 Hz, H-4b), and 4.72 (m, H-3).

**Table 1:** ^1^H-(400 MHz) and ^13^C-NMR (100 MHz) data of compounds **1** and **2** in CD_3_OD (*δ* in ppm).

Position	1		2	
	*δ*_H_ (*J* in Hz)	*δ*_C_	*δ*_H_ (*J* in Hz)	*δ*_C_
1		171.4, s		140.4, s
2			7.26 (overlap, 1H)	129.3, d
3	4.72 (m, 1H)	77.8, d	7.24 (overlap, 1H)	129.9, d
4	3.46 (dd, *J* = 17.0, 3.3 Hz, 1H)	29.4, t	7.19 (overlap, 1H)	127.2, d
	2.69 (dd, *J* = 17.0, 11.7 Hz, 1H)			
5		146.7, s	7.24 (overlap, 1H)	129.9, d
6	6.83 (d, *J* = 9.2 Hz, 1H)	116.7, d	7.26 (overlap, 1H)	129.3, d
7	7.45 (d, *J* = 9.2 Hz, 1H)	126.7, d	2.86 (t, *J* = 6.7 Hz, 2H)	37.1, t
8		158.5, s	3.85 (dt, *J* = 9.7, 6.9 Hz, 1H)	69.4, t
			3.63 (dt, *J* = 9.7, 6.7 Hz, 1H)	
9		130.6, s		
10		109.5, s		
11	1.52 (d, *J* = 6.3 Hz, 3H)	21.1, q		
1''		172.6, s		
2''	1.98 (s, 3H)	20.7, q		
1'	5.35 (d, *J* = 3.7 Hz, 1H)	100.5, d	4.65 (d, *J* = 1.5 Hz, 1H)	101.5, d
2'	3.60 (dd, *J* = 9.7, 3.7 Hz, 1H)	73.2, d	3.77 (dd, *J* = 3.3, 1.7 Hz, 1H)	72.2, d
3'	3.82 (m, 1H)	74.8, d	3.60 (dd, *J* = 5.9, 3.2 Hz, 1H)	72.4, d
4'	3.36 (dd, *J* = 10.0, 8.9 Hz, 1H)	71.9, d	3.35 (d, *J* = 9.2 Hz, 1H)	73.8, d
5'	3.87 (m, 1H)	72.1, d	3.40 (dd, *J* = 9.4, 6.0 Hz, 1H)	69.7, d
6'	4.36 (dd, *J* = 11.8, 2.1 Hz, 1H)	64.9, t	1.19 (d, *J* = 6.0 Hz, 3H)	17.9, q
	4.18 (dd, *J* = 11.9, 6.7 Hz, 1H)			

**Figure 2:**
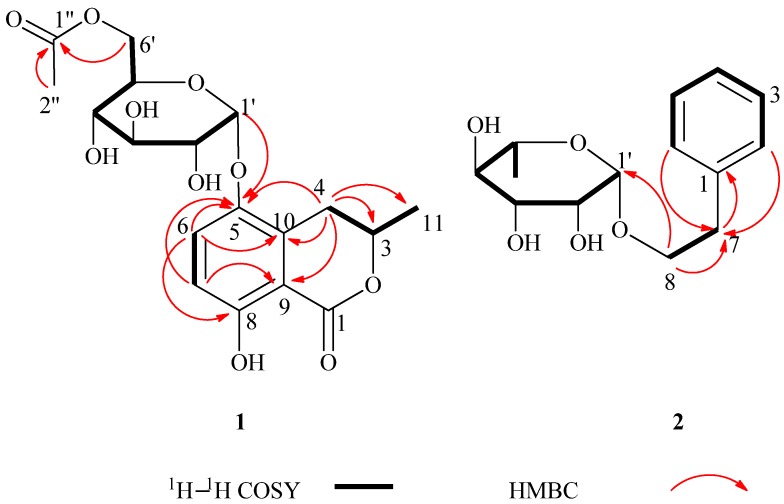
Key ^1^H–^1^H COSY and the selected HMBC correlations of compounds **1** and **2**.

Furthermore, the HMBC correlations of H-4 (*δ*_H_ 2.46, 2.69) with C-3 (*δ*_C_ 77.8), C-5 (*δ*_C_ 146.7), C-9 (*δ*_C_ 130.6), and C-10 (*δ*_C_ 109.5); H-6 (*δ*_H_ 6.83) with C-5 (*δ*_C_ 146.7), C-8 (*δ*_C_ 158.5), and C-10 (*δ*_C_ 109.5); H-7 (*δ*_H_ 7.45) with C-5 (*δ*_C_ 146.7), C-8 (*δ*_C_ 158.5), and C-9 (*δ*_C_ 130.6) in the HMBC spectrum as well as the spin systems in the ^1^H–^1^H COSY spectrum (*δ*_H_ 1.52/4.72, *δ*_H_ 4.72/2.69, and *δ*_H_ 6.83/7.45) indicated the presence of a dihydroisocoumarin skeleton (Figure 1) [7,8]. In the HMBC spectrum of **1**, the key HMBC correlation of H-1' (*δ*_H_ 5.35) with C-5 (*δ*_C_ 146.7) implied that the sugar unit was located at C-5 of the dihydroisocoumarin skeleton. In addition, the correlation of H-6' (*δ*_H_ 4.36, 4.18) to C-1' (*δ*_C_ 172.6) indicated that the acetyl group (*δ*_C_ 172.6, 20.7) was located at C-6' of the hexosyl sugar moiety (Figure 2). After hydrolysis of **1** with 4 M aqueous CF_3_COOH the sugar unit was confirmed to be *α*-d-glucose [9], as determined by GC analysis of its trimethylsilylated derivative and the coupling constant of its anomeric proton (*J* = 3.7 Hz) [10]. The linkage of the d-glucose to the dihydroisocoumarin skeleton was unambiguously established by the HMBC experiment that showed cross-peaks between *δ*_H_ 5.35 (H-1_glc_) and *δ*_C_ 146.7 (C-5).

Compound **1** has a CD spectrum that similar to that of (3*R*)-5-hydroxymellein with negative extrema at 226 and 255 nm [8]. Thus, compound **1** was identified to be 3*R*-(+)-5-*O*-[6'-*O*-acetyl]-*α*-d-glucopyranosyl-5-hydroxymellein.

Compound **2** was obtained as a colorless gum with the molecular formula C_14_H_20_O_5_, as deduced from its HRESIMS peak at *m/z* 291.1200 [M+Na]^+^ (calcd. 291.1203). 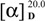 −46 (*c* = 4.83, CH_3_OH). The IR spectrum of **2** showed absorptions due to hydroxyl (3398 cm^−1^) and aromatic (1452 and 1497 cm^−1^) functionalities. The ^1^H-NMR data of **2** (Table 1) showed a set of monosubstituted aromatic ring signals at *δ*_H_ 7.15–7.28 (5H, overlapped), and a methyl doublet at *δ*_H_ 1.19 (d, *J* = 6.0 Hz, 3H), and oxygenated proton at *δ*_H_ 4.65 (d, *J* = 1.5 Hz, 1H). The ^13^C-NMR and DEPT showed six aromatic carbons (one quaternary carbon), two methylenes (one oxygenated), five methine carbons (three oxygenated and one anomeric), and one methyl group. According to the signals of six aromatic carbons and ^1^H–^1^H COSY signal at H-8 (*δ*_H_ 3.63, 3.85) with H-7 (*δ*_H_ 2.86) indicated compound **2** was a phenylethanol derivative. A series of proton signals in the range of *δ*_H_ 3.3–3.8, 4.65 (d, *J* = 1.5 Hz, 1H), and 1.19 (d, *J* = 6.0 Hz, 3H), and their corresponding carbons at *δ*_C_ 69.7, 72.2, 72.4, 73.8, 101.5, and 17.9 were observed in the ^1^H- and ^13^C-NMR spectra, which indicated the existence of a *α*-rhamnose moiety. After acid hydrolysis of **2**, the sugar moiety was identified to be *α*-l-rhamnose according to the GC analysis. In the HMBC spectrum, the correlation of H-8 (*δ*_H_ 3.63, 3.85) with C-1' (*δ*_C_ 101.5) showed that the *α*-rhamnose was attached to C-8 (Figure 2), hence compound **2** was identified as (−)-phenylethyl-8-*O*-*α*-l-rhamnopyranoside.

The known compounds **3**–**7** were identified as *β*-sitosterol (**3**) [11], stigmast-4-en-3-one (**4**) [12], ergosterol (**5**) [13], (22*E*)-cholesta-4,6,8(14),22-tetraen-3-one (**6**) [14] and 4*α*-methylergosta-8(14),24(28)-dien-3*β*-ol (**7**) [15], respectively, by comparison of their spectroscopic data with literature values.

## 3. Experimental

### 3.1. General Procedures

Optical rotations were measured on a Perkin-Elmer PE-341LC polarimeter (PerkinElmer, Waltham, MA, USA). UV spectra were recorded on a PerkinElmer Lambda 35 spectrophotometer (PerkinElmer, Waltham, MA, USA). IR spectra were recorded on a Bruker VERTEX 70 spectrometer (Bruker, Ettlingcn, Germany). CD spectrum was detected on Jasco J-810 spectrometer (Jasco, Hachioji, Japan). 1D and 2D NMR spectra were recorded on a Bruker AM-400 NMR spectrometer (Bruker, Ettlingcn, Germany) using CD_3_OD (*δ*_H_ 3.31/*δ*_C_ 49.0) signals as standard, and chemical shifts were recorded as values. HRESIMS data were acquired using a Thermo Fisher LC-LTQ-Orbitrap XL spectrometer (Thermo Fisher, Waltham, MA, USA). TLC was carried out using glass-precoated silica gel GF_254_ (Qingdao Marine Chemical, Inc., Qingdao, China) and visualized under UV light or by spraying with vanillin (contains H_2_SO_4_) ethanol reagent. Silica gel (100−200 mesh and 200−300 mesh, Qingdao Marine Chemical Inc.), ODS (50 μm, YMC, Kyoto, Japan), and Sephadex LH-20 (Pharmacia Biotech AB, Uppsala, Sweden) were used for column chromatography. Semi-preparative HPLC was performed on an Agilent 1100 liquid chromatography (Agilent, Santa Clara, CA, USA) with an YMC (10 × 250 mm, 5 μm) column. GC analysis was performed with a GC-14CPTF gas chromatography system (Shimadzu, Shimane, Japan) with an Agilent Innowax capillary column.

### 3.2. Fungal Material and Aphylogenetic Analysis of ITS 1–4 Gene Sequence

The strain of the fungus obtained from China Forestry Culture Collection Center (CFCC, Beijing, China) was isolated from *Pinus tabuliformis* (altitude: 789 m; longitude: 108°; latitude: 33°) by Xiaobin Song (Associate Researcher at the College of Forestry, Northwest A&F University) in Shanxi Province at August 2008. It is available to specialists from the CFCC, Preservation Serial number: cfcc 87468. A voucher specimen of the culture (No. 2013–0820) was deposited in the herbarium of Hubei Key Laboratory of Natural Medicinal Chemistry and Resource Evaluation, Tongji Medical College, Huazhong University of Technology and Science, Wuhan, China.

Fungal genomic DNA was extracted by the CTAB method [16]. ITS gene fragments were amplified by general primers ITS1 (5'-TCCGTAGGTGAACCTGCGG-3') and ITS4 (5'-TCCTCCGCTTATTGA TATGC-3'). The PCR conditions used were as follows: initial denaturation at 94 °C for 5 min, followed by 35 cycles of 94 °C for 1 min, 55 °C for 40 s, 72 °C for 1 min, and a final extension at 72 °C for 10 min. PCR reaction mixtures (20 μL) contained 100 ng genomic DNA, 2 μL 10 × PCR reaction buffer, 2 µL 10 μM MgCl_2_, 0.5 μL 10 μM forward and reverse primers, 0.5 μL deoxyribonucleotide triphosphate (2.5 μM each), and 0.3 μL 5 U of Taq DNA polymerase. All the reagents for sequencing were from Hubei Bios Biological Technology Co, Ltd, Wuhan, China. The amplified products were sequenced and aligned with the sequences in GenBank by the BLASTN program. The results showed that the gene sequences of fungus were belonging to the *Xylaria* sp.

The closest matches in Genbank were obtained from sequences that were declared to be “*Xylaria* sp”. However, the highest homology with a properly identified species was that of *Nemania diffusa*. The corresponding sequence showed 98% homology in BLAST [17]. Therefore, the identity of this fungus with a member of the genus *Xylaria* is not absolutely certain. As outlined by Stadler *et al*. [18], much work remains to be done until the endopyhtic *Xylariaceae* can be identified on the basis of ITS DNA sequences. The gene sequence of *Xylaria* sp. cfcc 87468 has been deposited in GenBank, with GenBank accession number KJ 139985.

### 3.3. Fermentation and Isolation

The fungus *Xylaria* sp. cfcc 87468 maintained in potato dextrose agar (PDA) was directly inoculated on plates of nutrient agar media kept at 28 °C for 9 days. Fermentation was carried out in 30 Erlenmeyer flasks (500 mL), each containing 100 g of rice and 0.3% peptone. Distilled H_2_O (100 mL) was added to each flask, and the rice was soaked overnight before autoclaving at 121 °C under 15 psi for 30 min. After cooling to room temperature, each flask was inoculated with the fresh mycelium and incubated at 28 °C for 35 days. The fermented solid rice medium (3.0 kg) was soaked with ethyl acetate (6 L × 3, 2 days for each time) at room temperature. The solvent was evaporated *in vacuo* to afford a residue (32.0 g).

The crude residue (32.0 g) was subjected to silica gel (200−300 mesh) column chromatography, with a step gradient elution with petroleum ether–ethyl acetate (40:1→10:1→5:1→2:1→1:1→0:1) to yield four fractions (A–D). Fraction C (12.9 g) was chromatographed on an ODS column eluted with MeOH–H_2_O (70:30→0:100) to provide four subfractions (C_a_–C_d_). Subfraction C_c_ was further separated over Sephadex LH-20 eluting with CHCl_3_–MeOH (1:1) to give three subfractions (C_ca_–C_cc_). Subfraction C_ca_ was further purified by semi-preparative HPLC eluted with MeOH–H_2_O (100:0, flow rate: 2 mL/min) to give compounds **3** (5.8 mg, t_R_ 28 min) and **4** (6.0 mg, t_R_ 37 min), as well as compound **5** (2.7 mg, t_R_ 25 min) from subfraction C_cb_. Fraction D (2.7 g) was subjected to ODS column chromatography eluted with MeOH–H_2_O (50:50→0:1) to provide three subfractions (D_a_–D_c_). The subfraction D_a_ was further purified by semi-preparative HPLC eluted with MeOH–H_2_O (45:55, flow rate: 2 mL/min) to afford compounds **1** (9.2 mg, t_R_ 24 min) and **2** (38.0 mg, t_R_ 20 min). Fraction D_c_ was separated over Sephadex LH-20 eluting with CHCl_3_–MeOH (1:1), then subjected to semi-preparative HPLC eluted with MeOH–H_2_O (100:0, flow rate: 2 mL/min) to give compounds **6** (7.4 mg, t_R_ 45 min) and **7** (27.0 mg, t_R_ 39 min).

### 3.4. Hydrolysis and Determination of the Absolute Configuration of the Sugar Moiety

A solution of **1** (1.5 mg) in 4 M aqueous CF_3_COOH (2.0 mL) was heated at 100 °C for 3 h in a water bath. The reaction mixture was diluted in H_2_O (4.0 mL) and extracted with EtOAc (4.0 mL × 3), then the aqueous layer was concentrated to remove CF_3_COOH. The residue was dissolved in pyridine (1.0 mL), to which l-cysteine methyl ester hydrochloride (1.5 mg) in pyridine (1.0 mL) was added. Then, the mixture was kept at 60 °C for 2 h. The reaction mixture was concentrated to dryness and then trimethylsilylimidazole (0.2 mL) was added to the residue, followed by stirring at 60 °C for 1 h in a water bath. Finally, the mixture was partitioned between hexane and H_2_O (0.3/4.0 mL) and the hexane extract was analyzed by gas-chromatography (GC) under the following conditions: GC-14CPTF gas chromatography system; Agilent Innowax capillary column (30 m × 0.53 mm × 1.0 μm); column temperature, 205 °C; injection temp, 250 °C; detector FID, detector temp, 250 °C; carrier N_2_ gas; flow rate 2.5 mL/min; hydrogen flow, 25 mL/min; air flow, 250 mL/min; make up gas flow, 20 mL/min; injection volume, 2 μL. In compound **1**, d-glucose was confirmed by comparison of the retention times of the derivative with those of d-glucose and l-glucose derivatives prepared in a similar way, which showed retention times of 3.090 min and 3.632 min, respectively. As described above, the sugar in compound **2** was determined to be l-rhamnose with a retention time of 2.607 min.

### 3.5. Spectroscopic Data

*3R-(+)-5-O-[6'-O-Acetyl]-α-*d*-glucopyranosyl-5-hydroxymellein* (**1**). A colorless amorphous gum; 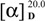 +60 (*c* = 1.33, CH_3_OH); CD (MeOH): λ_max_ nm (Δε)= 205.0 (+2.33), 209.0 (+2.24), 226.6 (−2.02), 255.0 (−3.75); UV (MeOH) *λ*_max_ (lg *ε*) 213 (4.47), 258 (3.70) nm; IR (film) *v*_max_ 3397, 2921, 1737, 1676, 1476, 1390, 1248, 1128, 1050, 910, 872 cm^−1^; ^1^H and ^13^C-NMR data, see Table 1; HRESIMS *m/z* 421.1097 [M + Na]^+^ (calcd for C_18_H_22_O_1__0_Na, 421.1105).

*(*−*)-Phenylethyl-8-O-α-*l*-rhamnopyranoside* (**2**). A colorless gum; 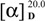 −46 (*c* = 4.83, CH_3_OH); UV (MeOH) *λ*_max_ (lg *ε*) 218 (2.85), 333 (2.13) nm; IR (film) νmax 3398, 2930, 1497, 1542, 1130, 1092, 1053, 981, 806, 749, 699 cm^−1^; ^1^H and ^13^C-NMR data, see Table 1; HRESIMS *m/z* 291.1200 [M+Na]^+^ (calcd for C_14_H_20_O_5_Na, 291.1203).

## 4. Conclusions

In this study, we investigated for the first time the chemical constituents of the ethyl acetate extract of *Xylaria* sp. cfcc 87468. Extensive spectroscopic analysis, chemical methods, and comparison with spectroscopic data in the literature resulted in the isolation of a new isocoumarin glycoside, 3*R*-(+)-5-*O*-[6'-*O*-acetyl]-*α*-d-glucopyranosyl-5-hydroxymellein (**1**), and a new phenylethanol glycoside, (−)-phenylethyl-8-*O*-*α*-l-rhamnopyranoside (**2**), along with five known steroids, *β*-sitosterol (**3**), stigmast-4-en-3-one (**4**), ergosterol (**5**), (22*E*)-cholesta-4,6,8(14),22-tetraen-3-one (**6**) and 4*α*-methylergosta-8(14),24(28)-dien-3*β*-ol (**7**). Compounds **3**–**7** were reported from *Xylaria* sp. cfcc 87468 for the first time.

## Supplementary Materials

Supplementary materials can be accessed at: http://www.mdpi.com/1420-3049/19/1/1250/s1.
