# Supplementary material for: Two New Secondary Metabolites from Xylaria sp. cfcc 87468
Source: Molecules. 2014 Jan 20;19(1):1250–7. doi: 10.3390/molecules19011250 (PMC6270958; doi:10.3390/molecules19011250)
Supplement: Supplementary file 1 [file molecules-19-01250-s001.pdf]

Supplementary Materials

Figure S1. HRESIMS spectrum of compound **1**.

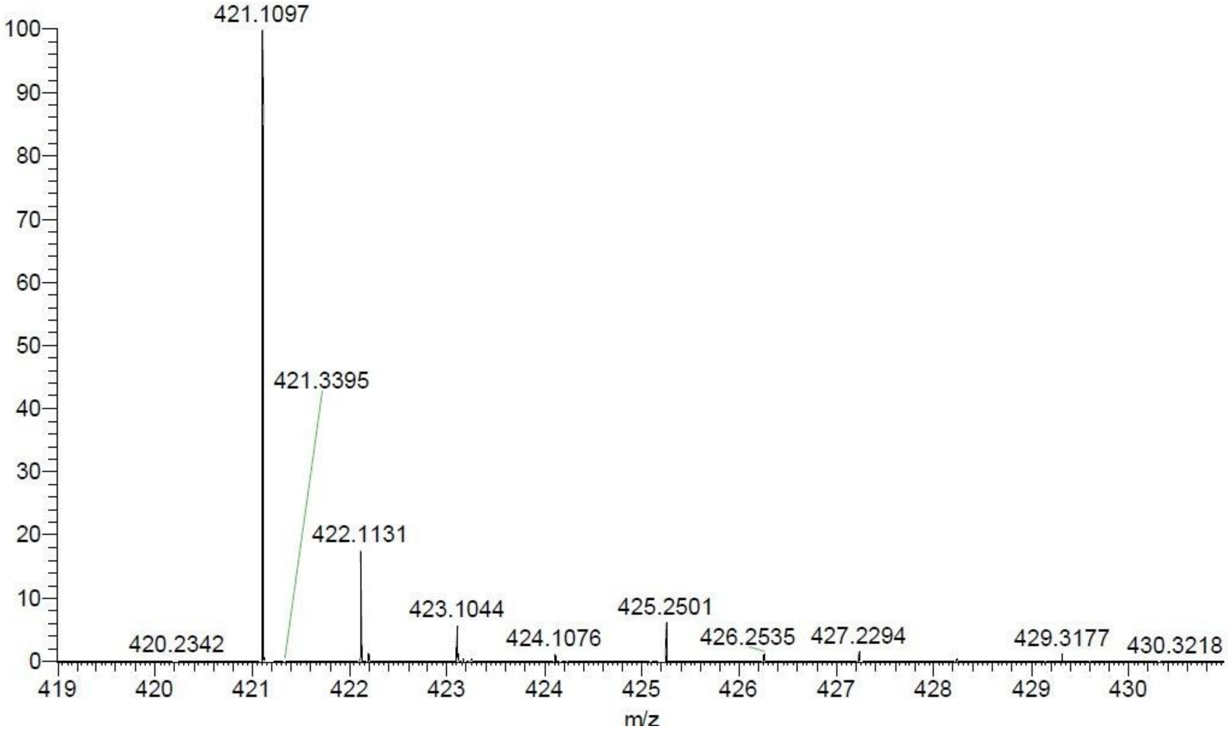

Figure S2. IR spectrum of compound **1**.

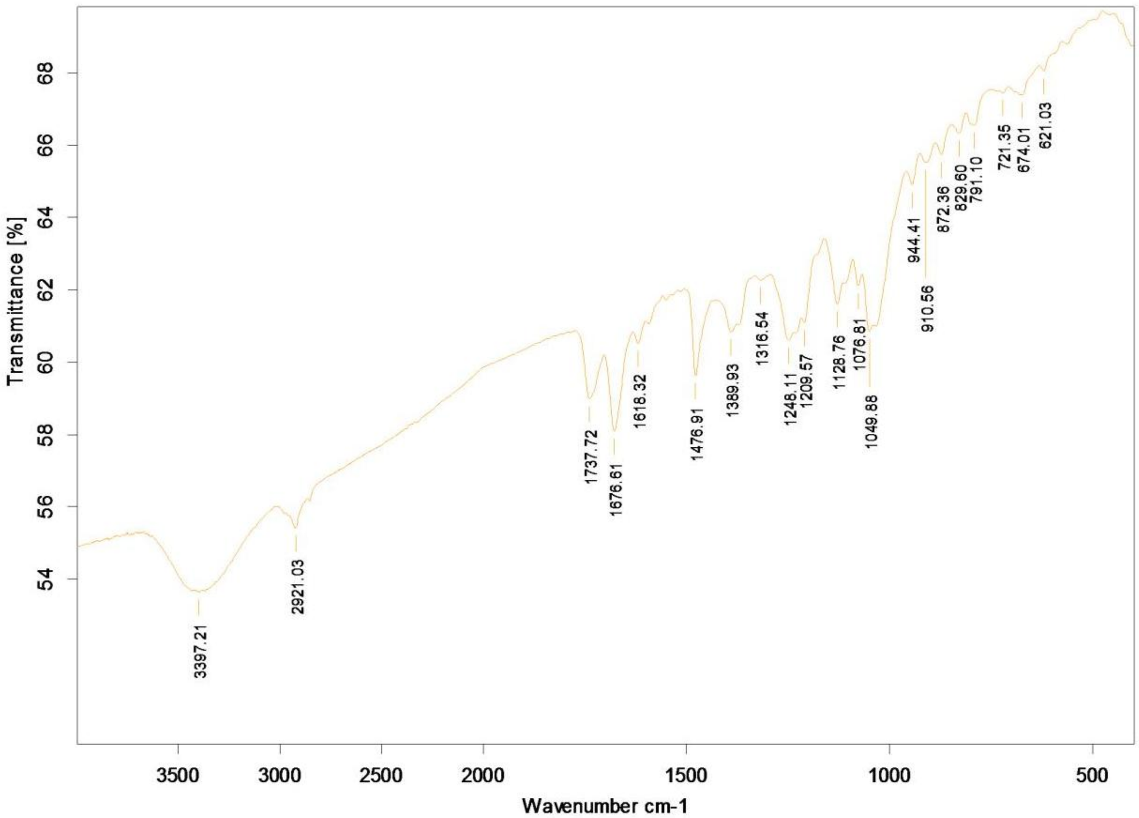

Figure S3. UV spectrum of compound **1**.

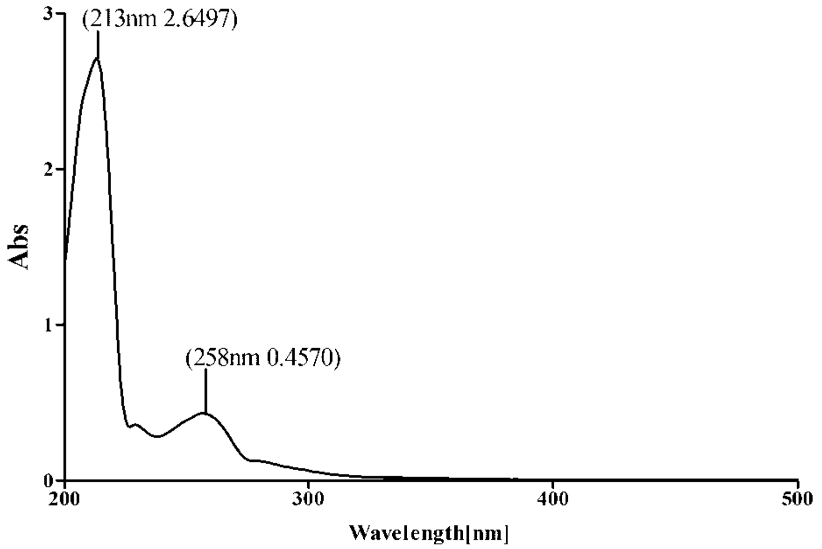

Figure S4. CD spectrum of compound **1**.

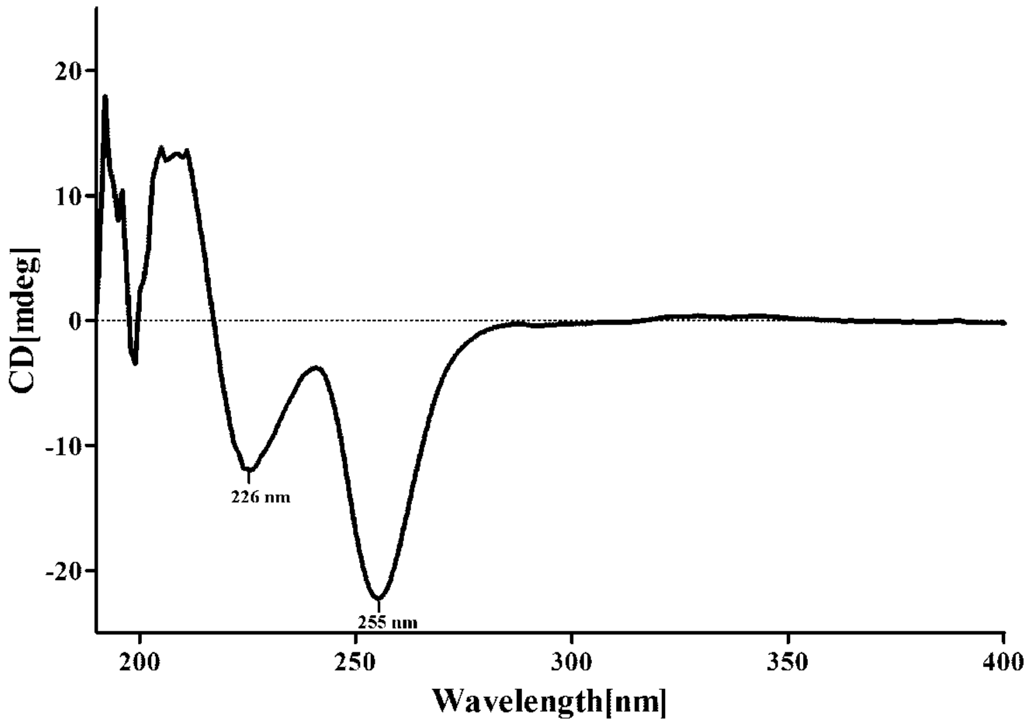

Figure S5. ^{1}H-NMR spectrum of compound **1**.

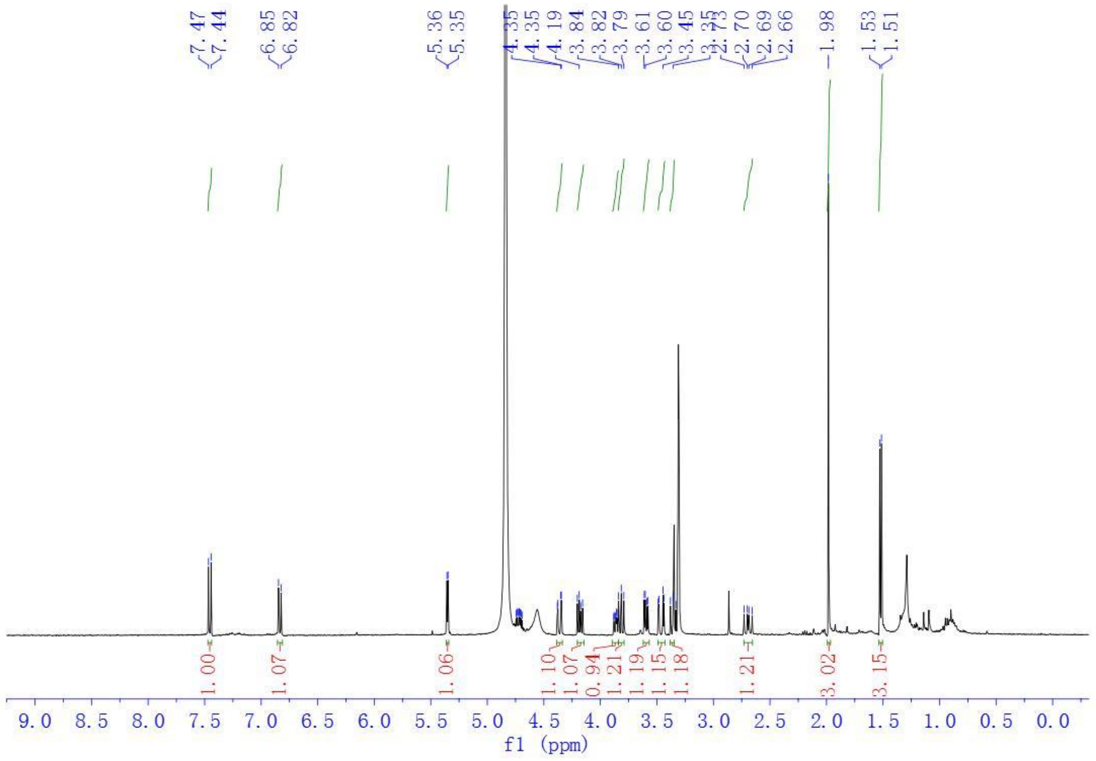

Figure S6. ^{13}C-NMR spectra of compound **1**.

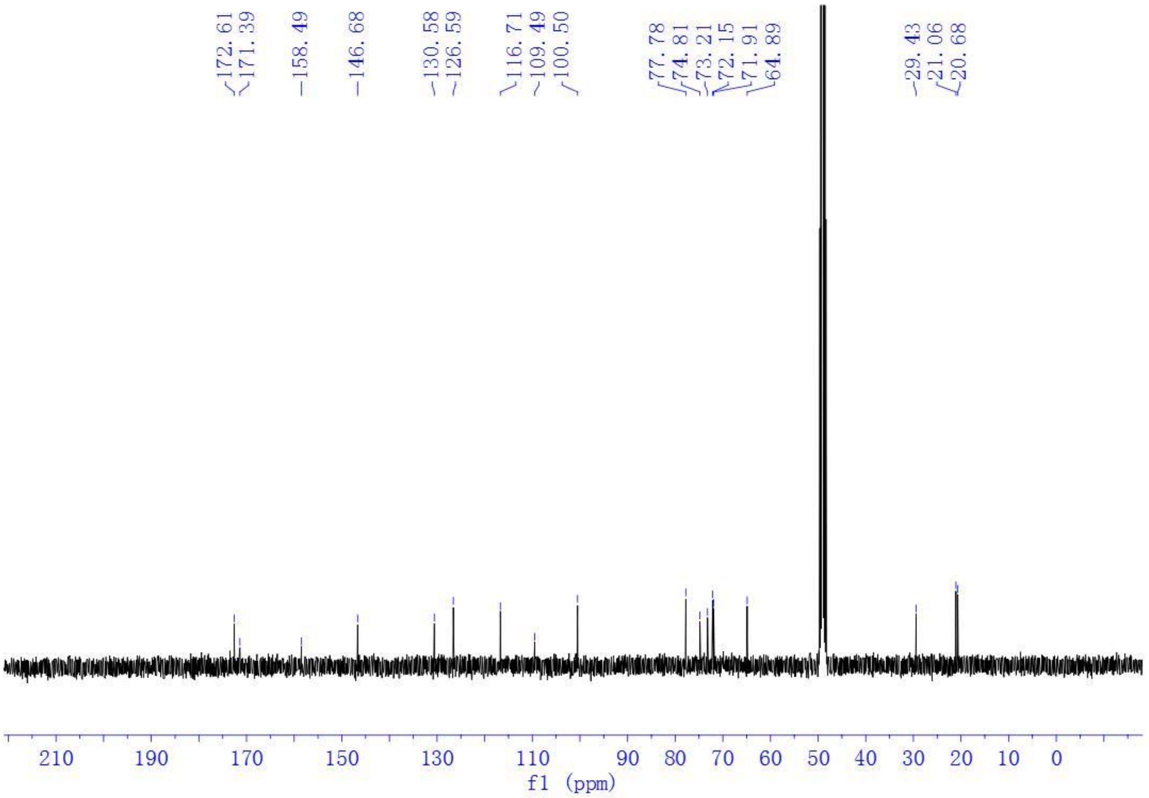

Figure S7. DEPT spectra of compound **1**.

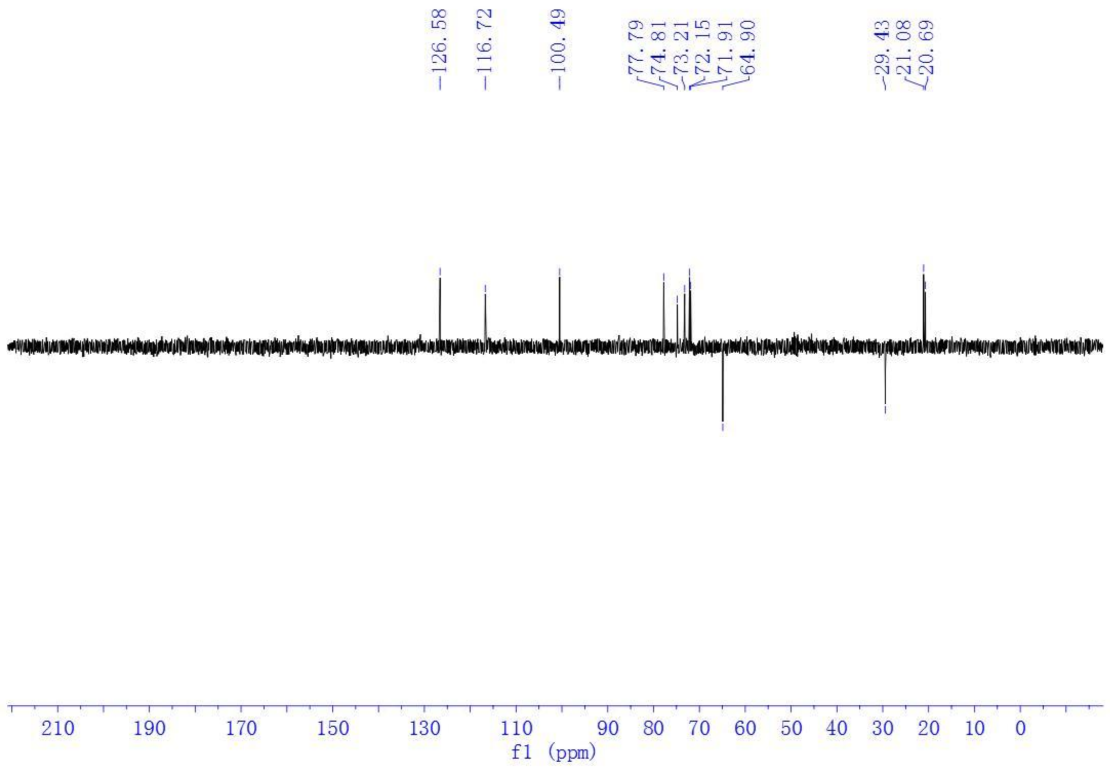

Figure S8. HSQC spectrum of compound **1**.

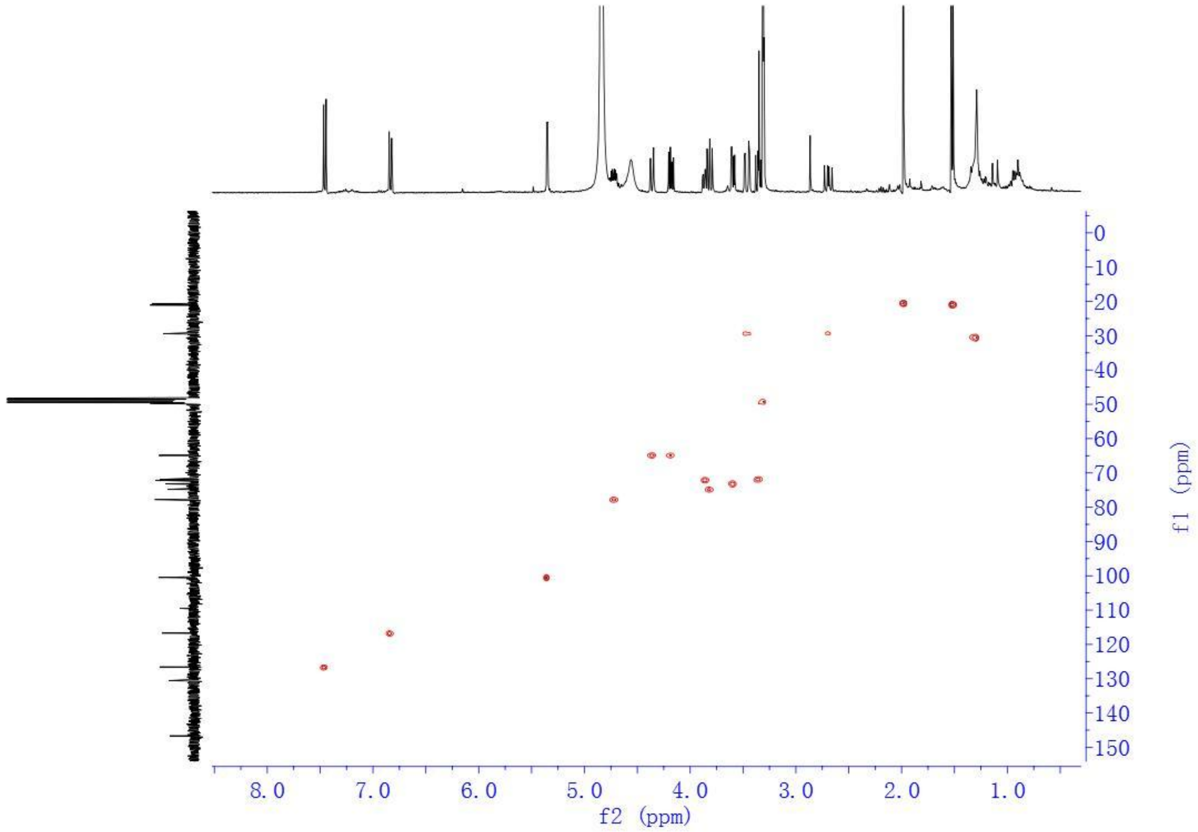

Figure S9. COSY spectrum of compound **1**.

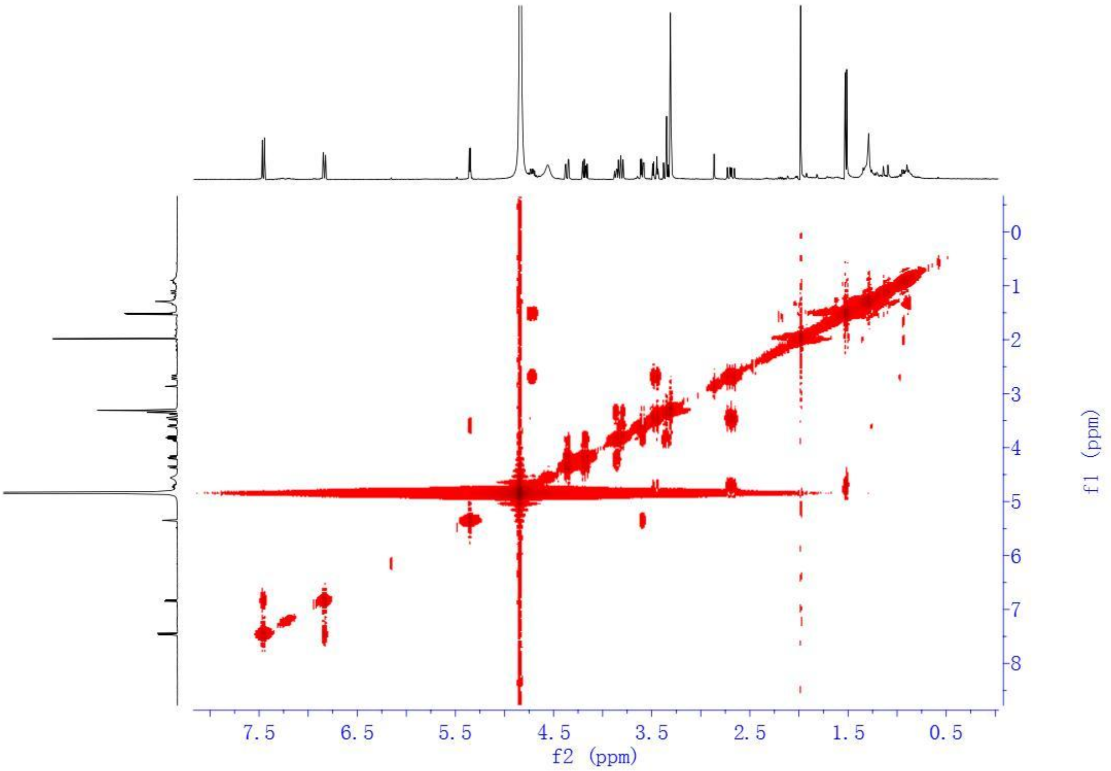

Figure S10. HMBC spectrum of compound **1**.

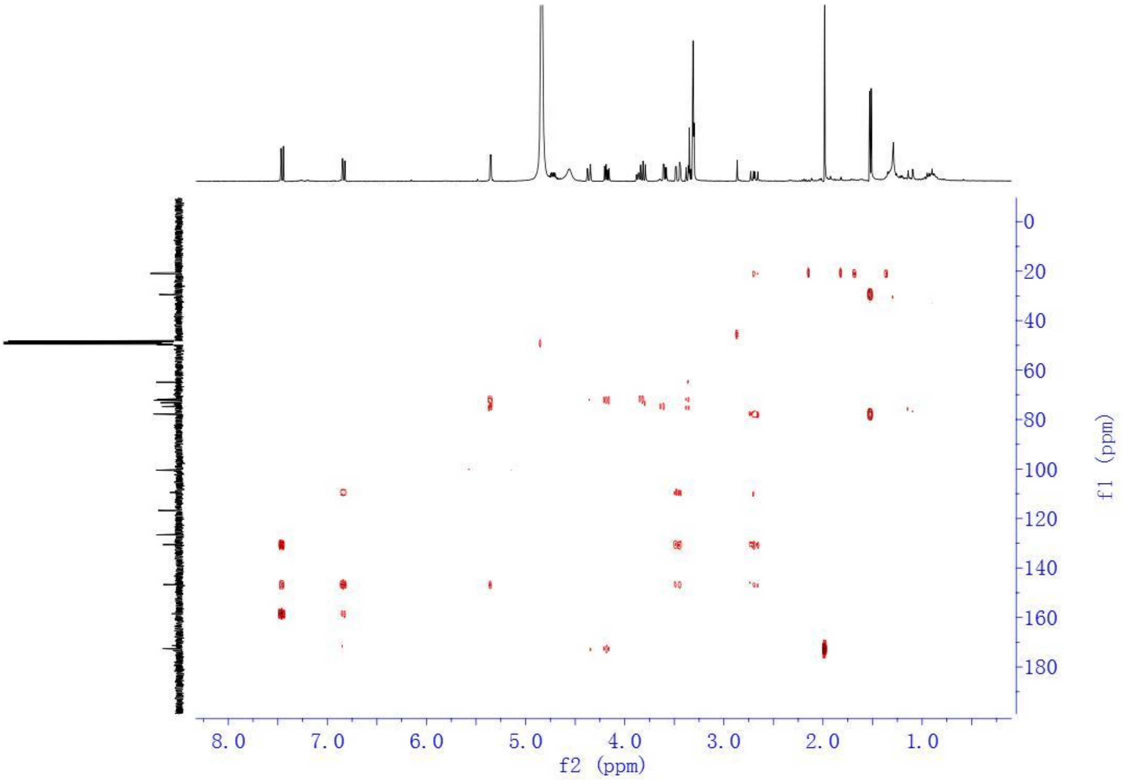

Figure S11. GS analysis of the sugar of compound **1** (**A**: L-glucose, t_R = 3.632 min; **B**: D-Glucose, t_R = 3.090 min; **C**: sugar of compound **1**, t_R = 3.090 min).

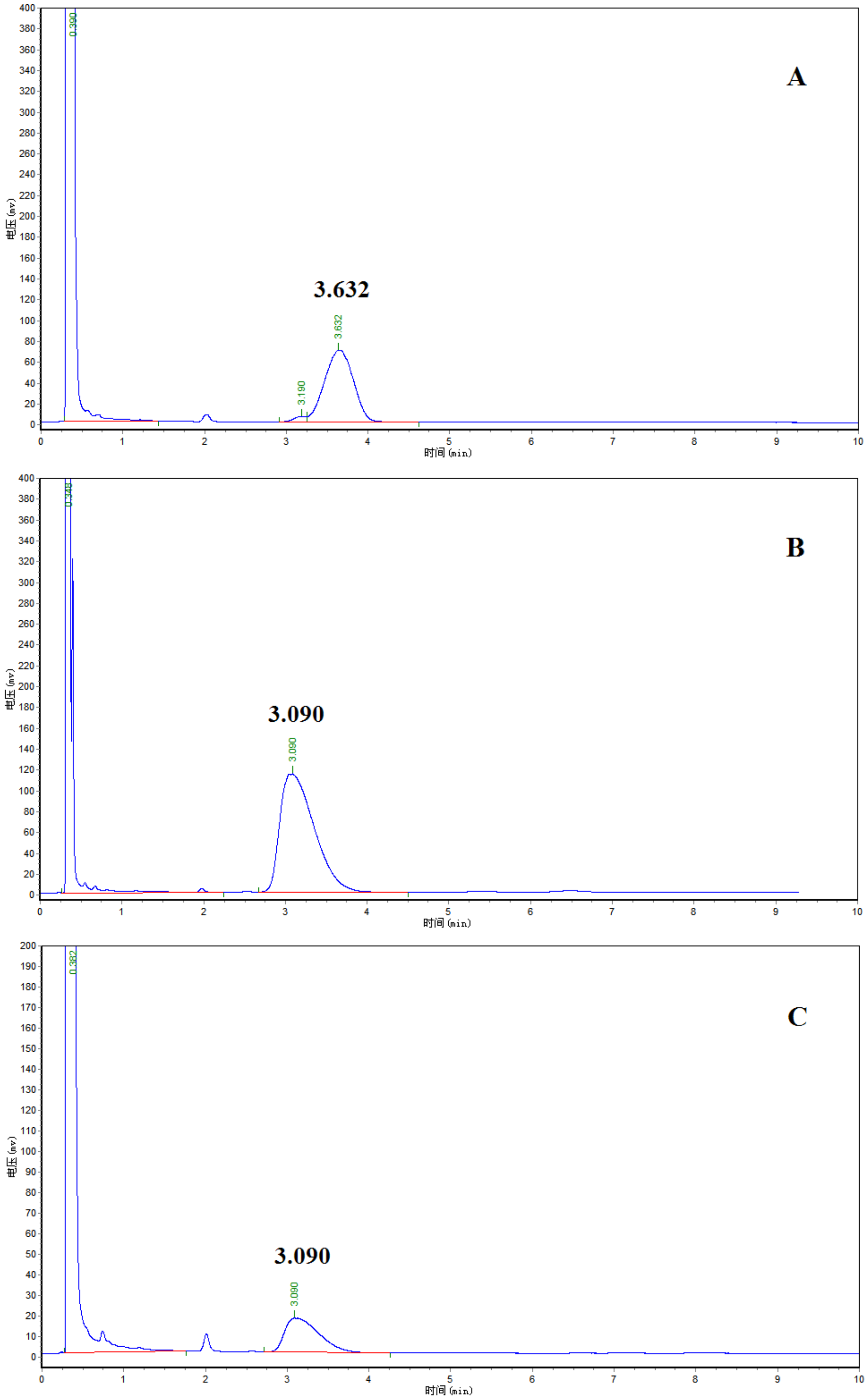

Figure S12. HRESIMS spectrum of compound **2**.

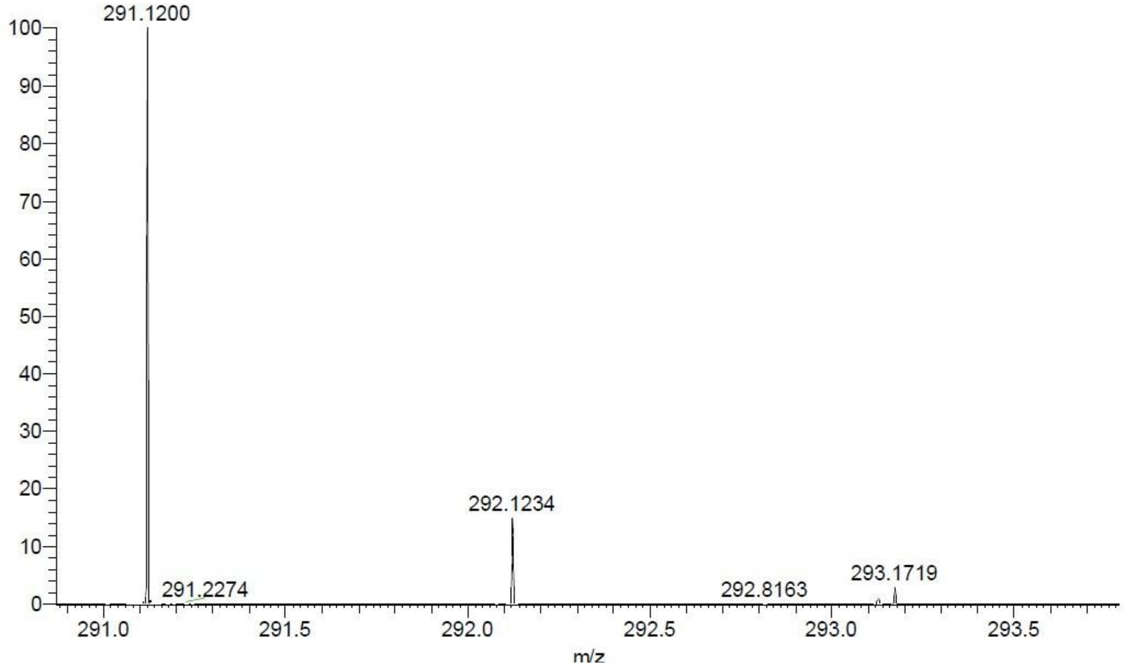

Figure S13. IR spectrum of compound **2**.

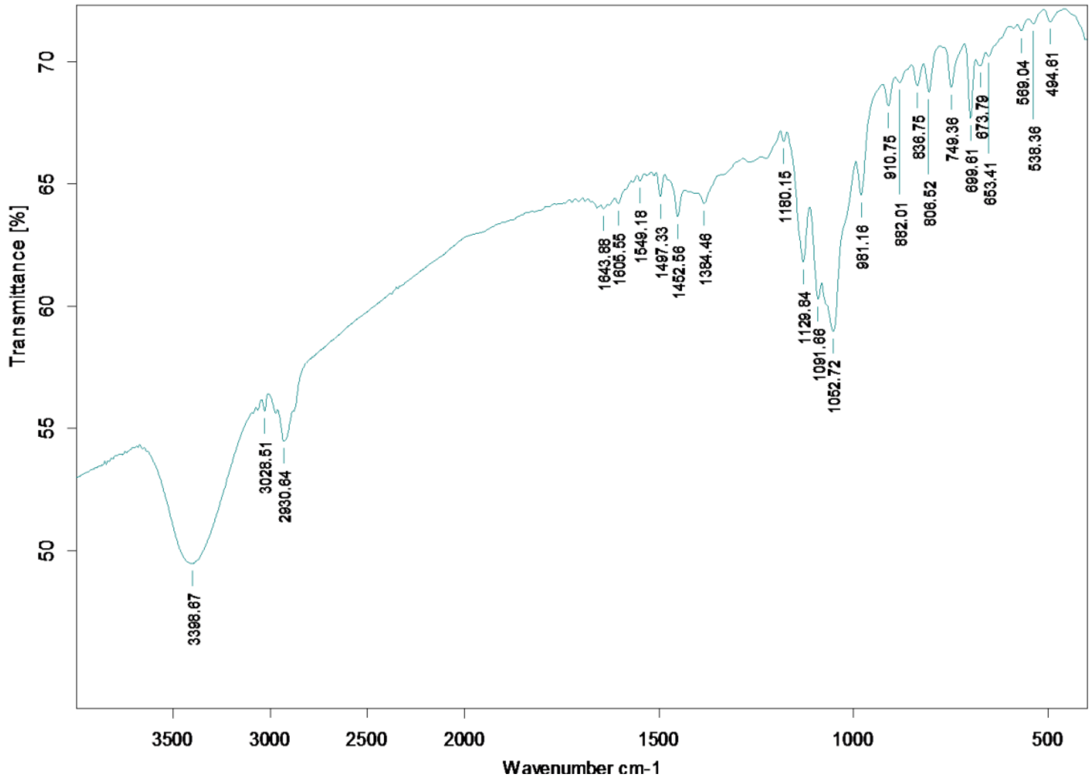

Figure S14. UV spectrum of compound **2**.

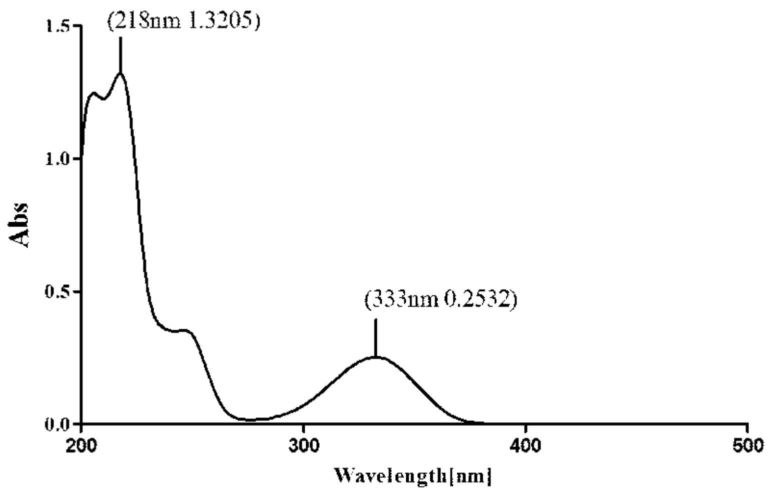

Figure S15. ^{1}H-NMR spectrum of compound **2**.

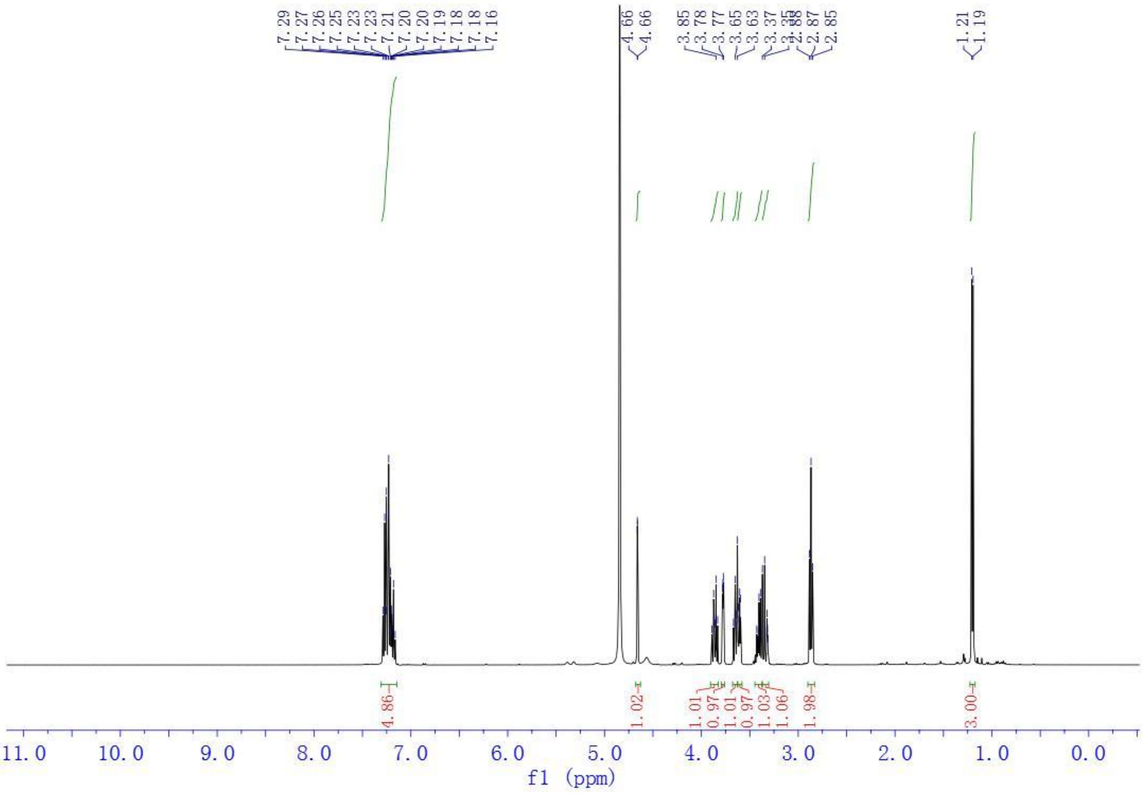

Figure S16. ^{13}C-NMR spectra of compound **2**.

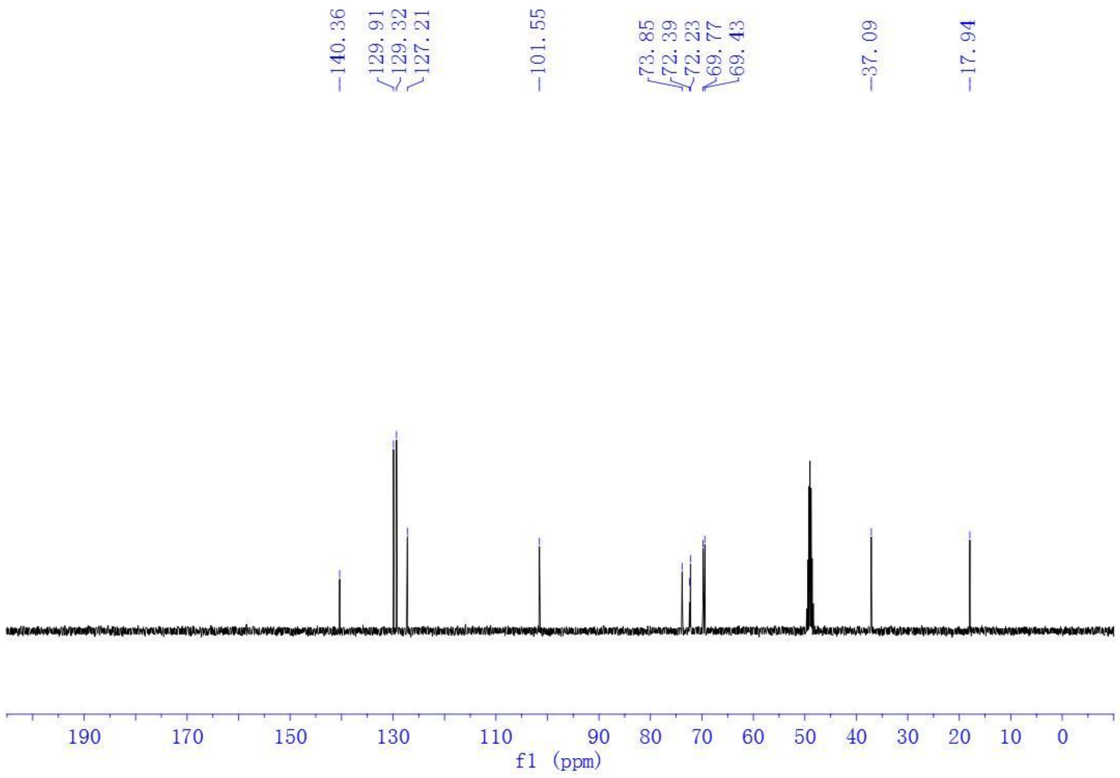

Figure S17. DEPT spectra of compound **2**.

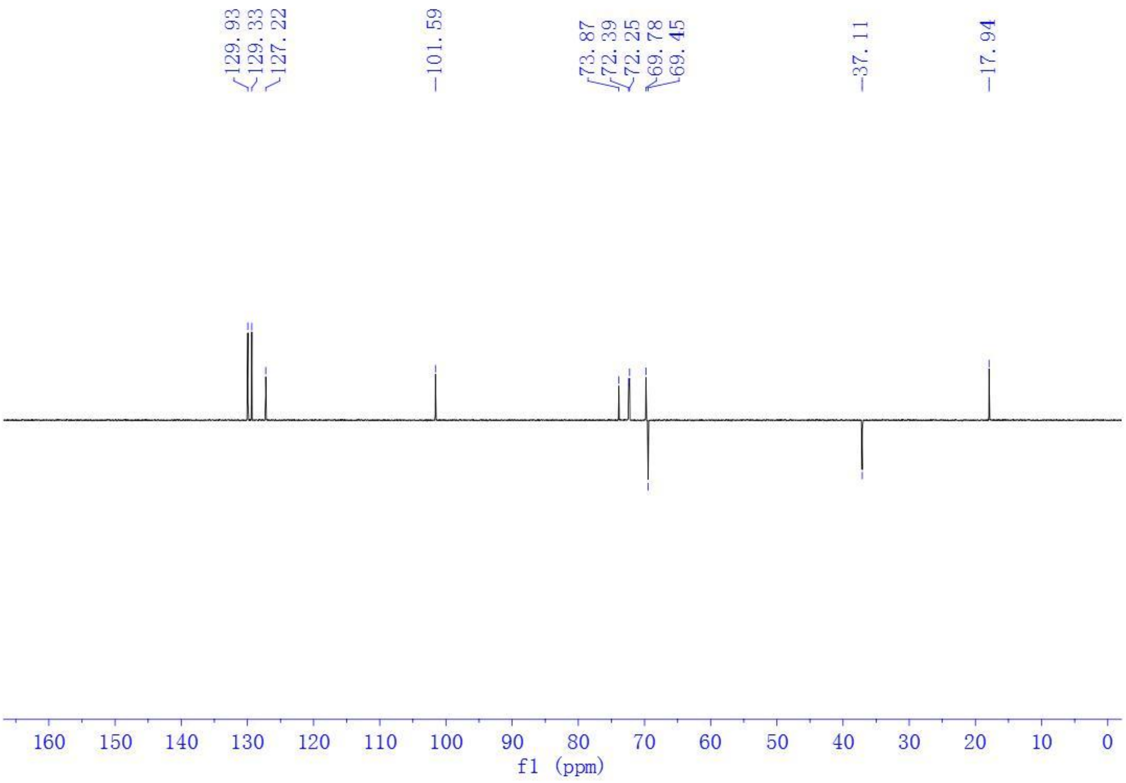

Figure S18. HSQC spectrum of compound **2**.

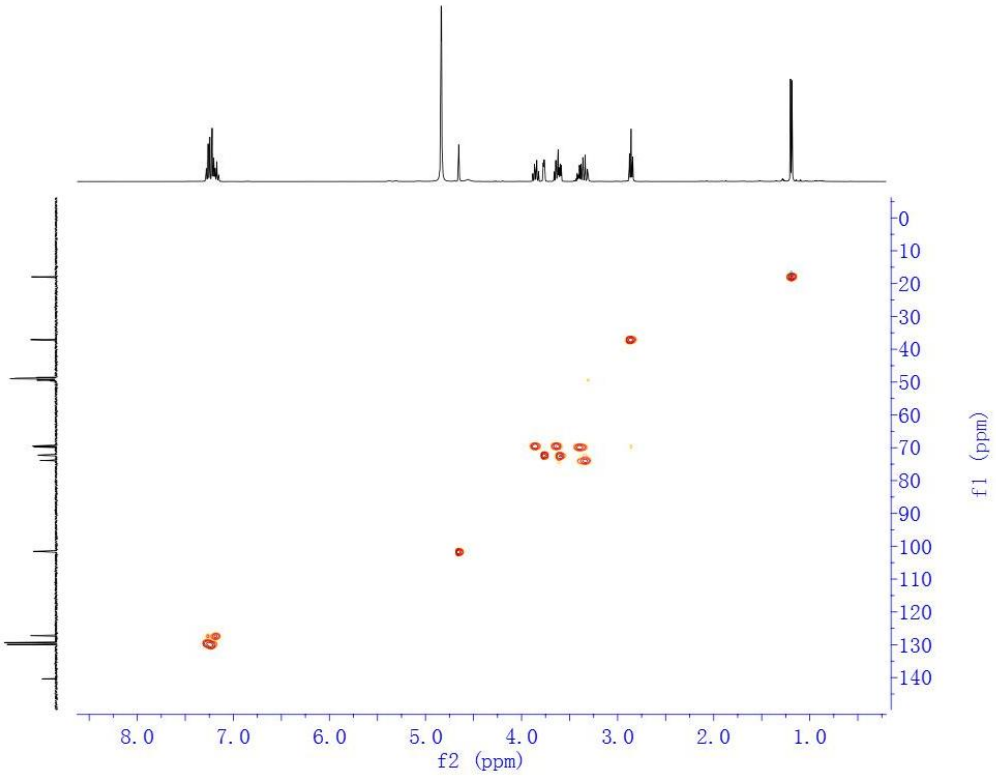

Figure S19. COSY spectrum of compound **2**.

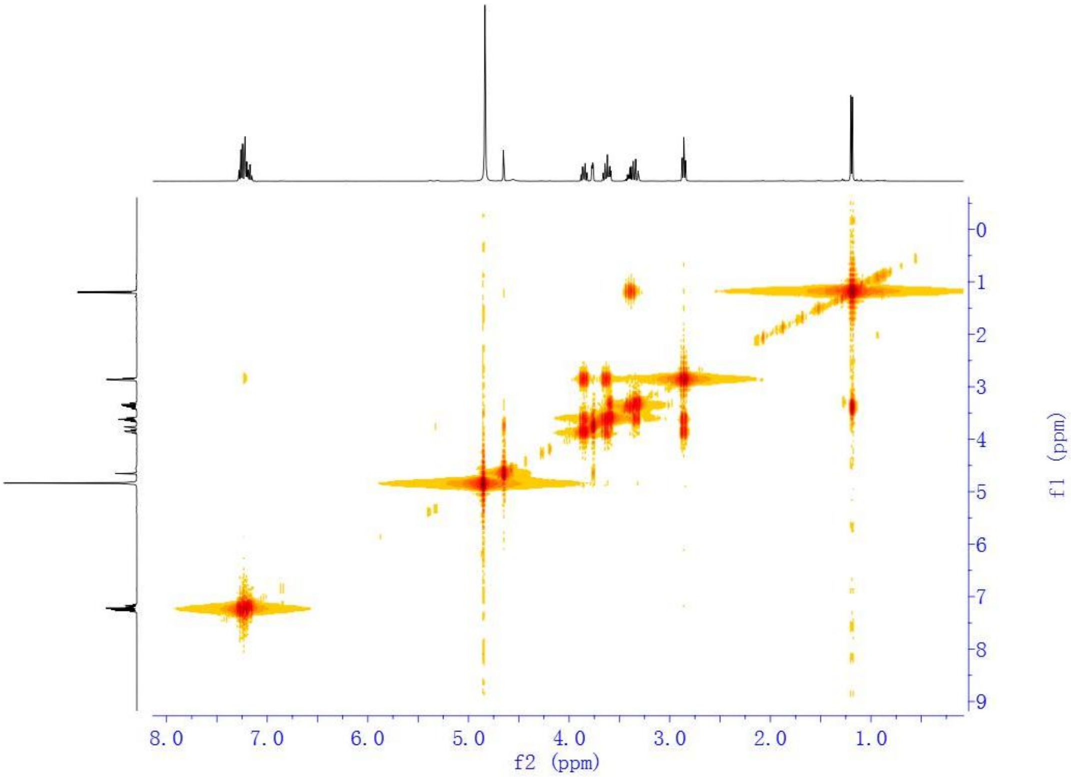

Figure S20. HMBC spectrum of compound **2**.

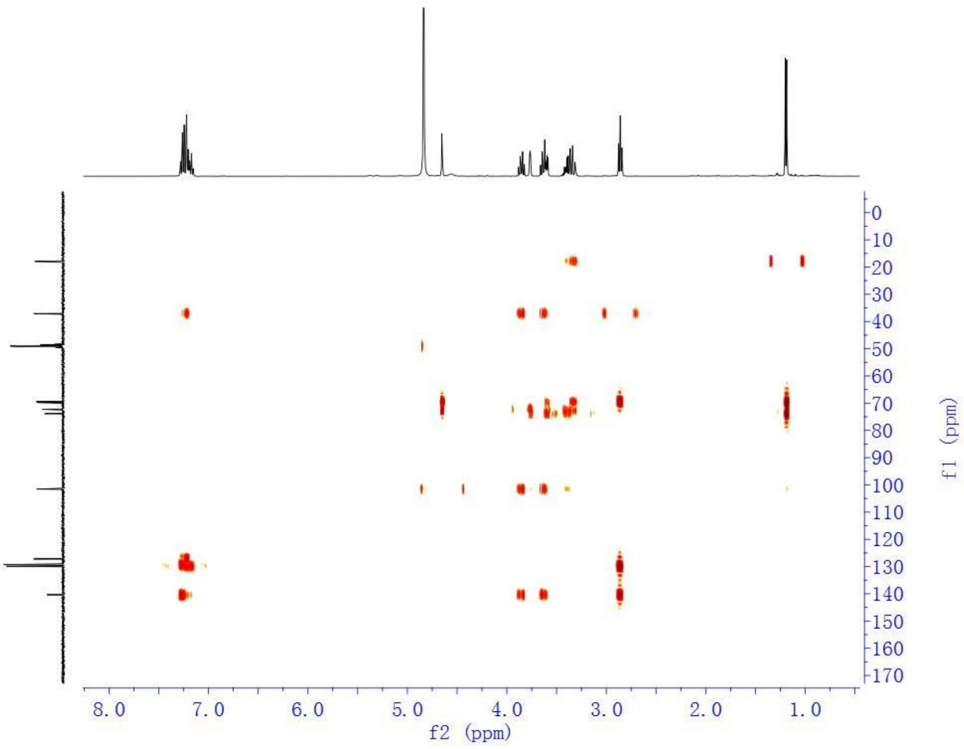

Figure S21. NOSEY spectrum of compound **2**.

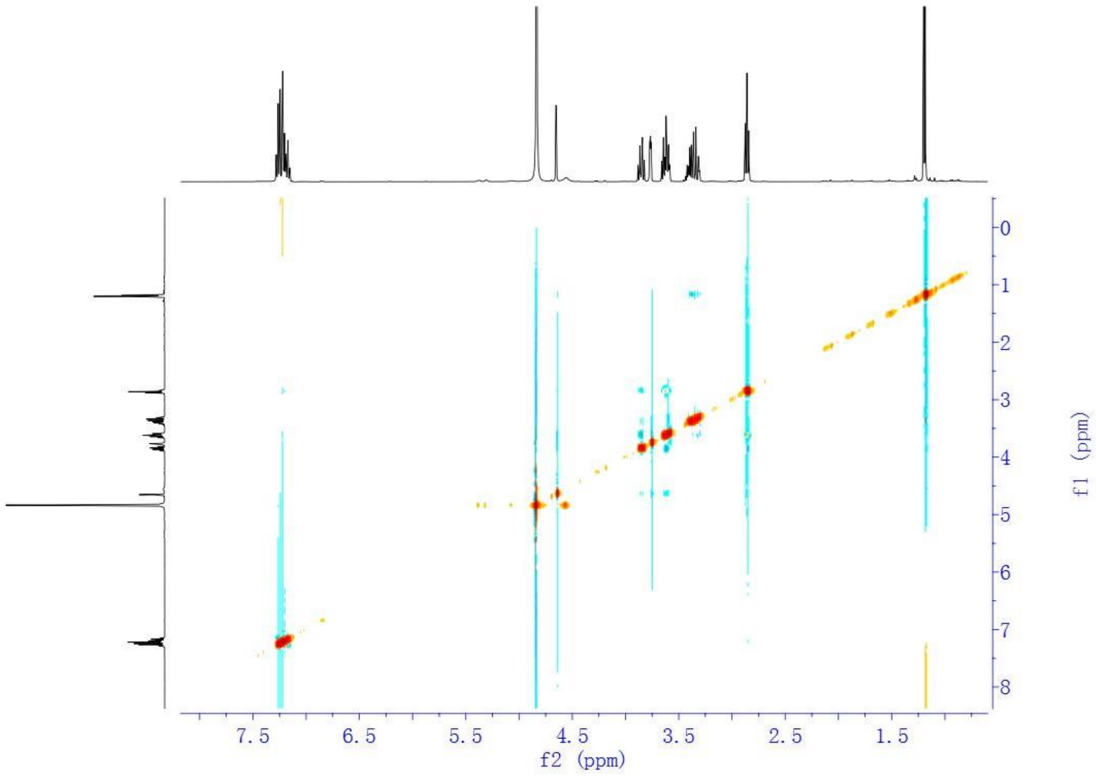

Figure S22. GS analysis of the sugar of compound **2** (**A**: L-rhamnose, t_R = 2.607 min; **B**: sugar of compound **2**, t_R = 2.573 min; **C**: A mixture 1:1 of L-rhamnose and sugar of compound **2**, t_R = 2.573 min).

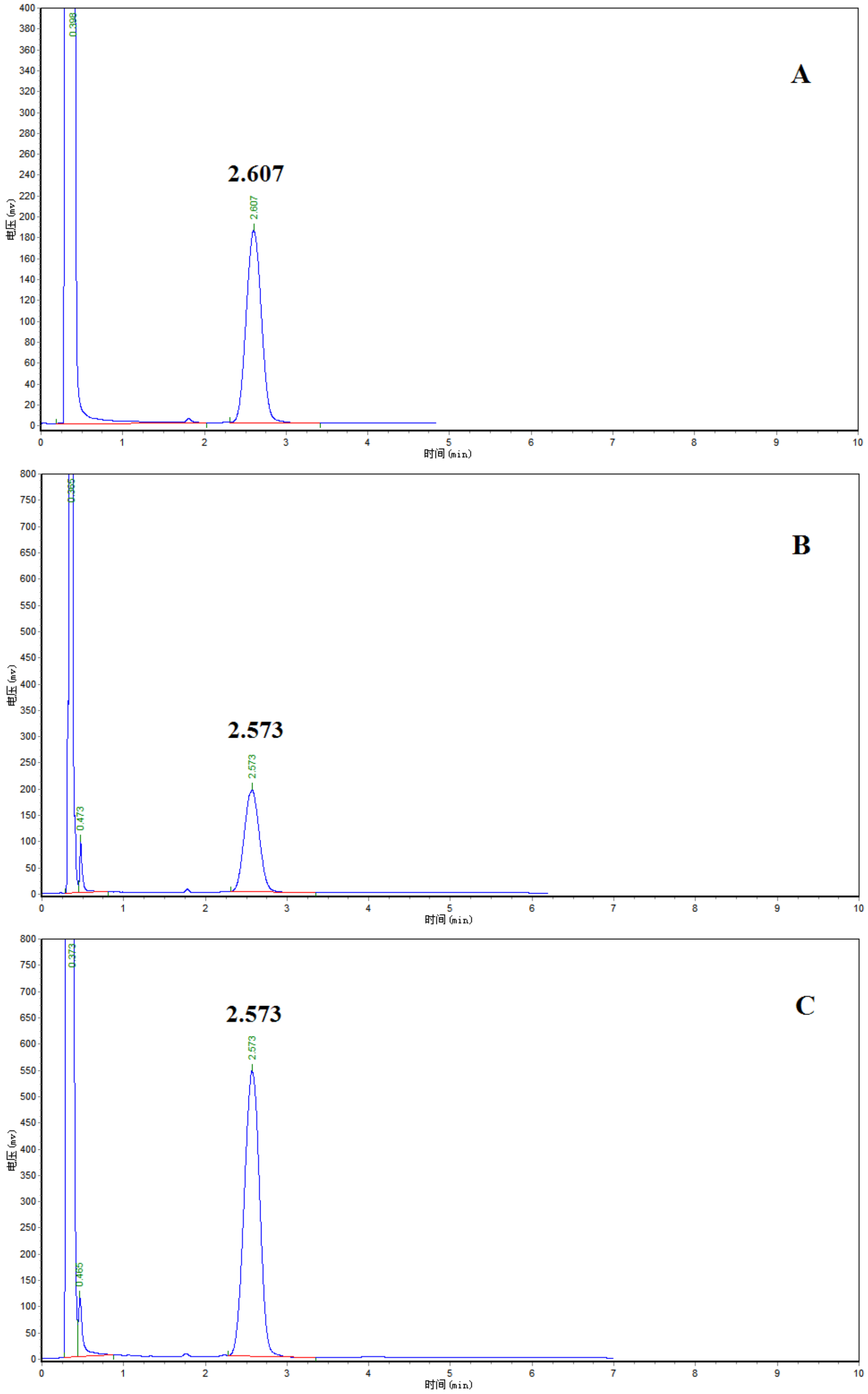

Figure S23. Pictures of the colony and the culture of *T. marneffei*.

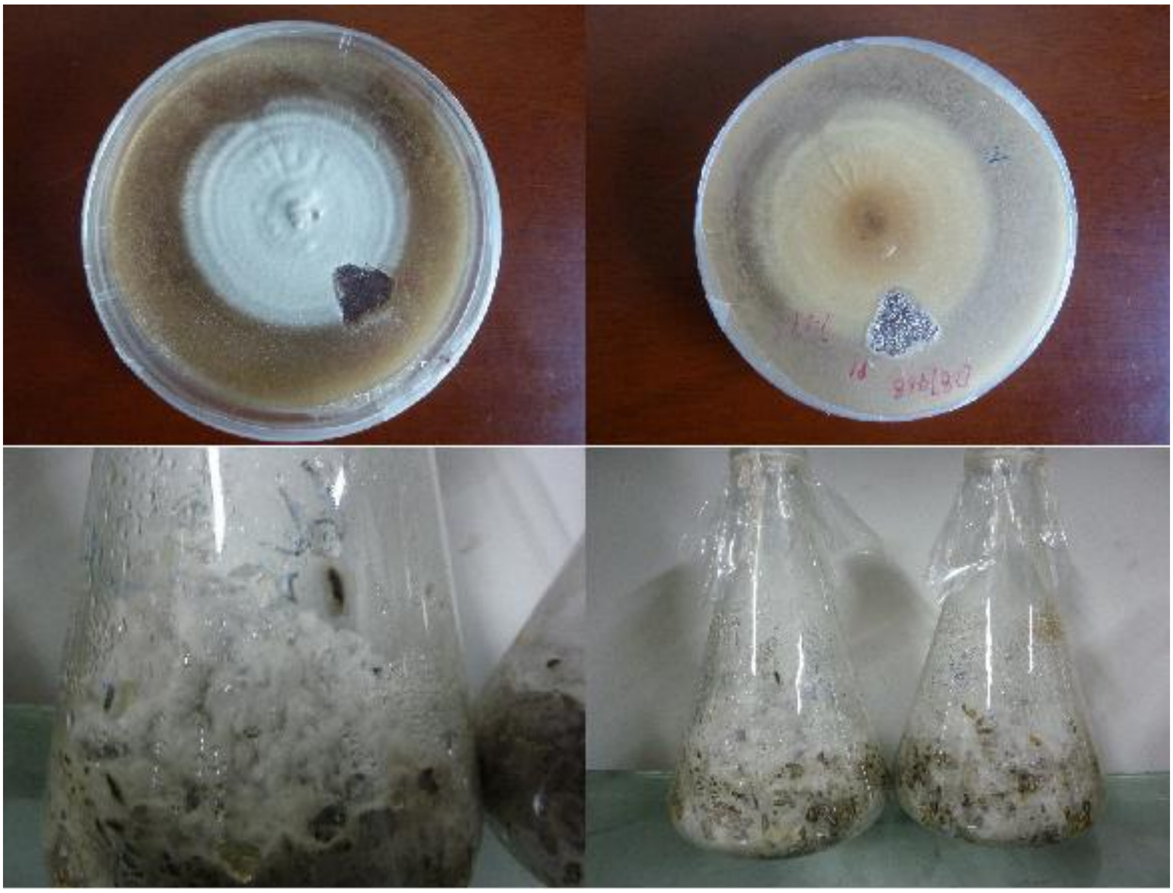

Figure S24. Phylogenetic analysis of ITS1-4 gene sequence.

GATGATGTATACTCCAACCCATGTGACATACCTCATGTTGCCTCGGCAGGTCGTGCCTCCCT
CGTAGGTCCTACCCTGTAGGCTCCTACCCGGAAGGCGCGGGTACCCCTGCCGGTGGCCCAG
GAAACTCTGTCTCATCGTTGAATTCTGAACCTATAACTAAATAAGTTAAAACTTTCAACAAC
GGATCTCTTGGTTCTGGCATCGATGAAGAACGCAGCGAAATGCGATAAGTAATGTGAATTG
CAGAATTCAGTGAATCATCGAATCTTTGAACGCACATTGCGCCCATTAGTATTCTAGTGGGC
ATGCCTGTTCGAGCGTCATTTCAACCCTTAAGCCCTTGTTGCTTAGCGTTGGGAGCCTACGG
CACCGTAGCTCCCCAAAGTCAGTGGCGGAGCCGGCTCACACTCTAGACGTAGTAATTTCTC
ACCTCGCCTATAGTTGGACCGGTCCCCTGCCGTAAAACGCCCCAGTATTTAAAAGGTTGAC
CTCGAATCAGGTAGGAATACCCGCTGAACTTAAGCATATCAATAAGCGGAGGA
